# Dosimetric evaluation of MobiusFX in the RapidArc delivery quality assurance comparing with 3DVH

**DOI:** 10.1371/journal.pone.0183165

**Published:** 2017-08-23

**Authors:** Ju-Young Song, Jae-Uk Jeong, Mee Sun Yoon, Sung-Ja Ahn, Woong-Ki Chung, Taek-Keun Nam

**Affiliations:** Department of Radiation Oncology, Chonnam National University Medical School, Gwangju, Korea; Calvary Mater Newcastle, AUSTRALIA

## Abstract

The dosimetric characteristics of MobiusFX, which uses the treatment machine log file to calculate the dose inside the patient body, were analyzed for use in the RapidArc delivery quality assurance (DQA) process. The accuracy and usefulness of MobiusFX in clinical cases was evaluated by comparing the dose calculated by MobiusFX with that calculated by the conventional measurement dose based program, 3DVH. The results of gamma evaluation with three different criteria (3%–3 mm, 4%–3 mm, 5%–3 mm) were analyzed, and the dose changes were calculated while simulating variable position errors (6 mm, 3 mm) and dosimetric output increases (6%, 3%). Although the doses calculated by each tool were not identical due to differences in the calculation algorithms, the doses calculated by MobiusFX were generally similar to those calculated by 3DVH. Based on these results, MobiusFX exhibited the required accuracy for clinical application. However, it could not determine the dosimetric output variation. It should therefore be considered a supplementary DQA tool that can verify the error in the daily treatment process, but not an ideal DQA tool that can replace conventional measurement based DQA methods.

## Introduction

Intensity modulated radiation therapy (IMRT), which can deliver a more appropriate dose to the tumor target while simultaneously decreasing the dose to the surrounding organs at risk (OAR), requires the delivery quality assurance (DQA) process in order to verify the dosimetric accuracy of the IMRT plan before being applied as patient treatment. Many studies have evaluated various DQA methods [1–5]. The conventional basic procedure for IMRT DQA can be summarized in two steps. The first is the measurement of the dose at a specific spatial point, while the second requires the acquisition of the dose distribution in a two-dimensional (2D) plane or three-dimensional (3D) space using film-irradiation or detector-array measurements. Then, errors are identified by comparing the measured data with data calculated using a treatment planning system (TPS). Although similar methods are used in the DQA process for volumetric arc therapy, such as RapidArc (Varian Medical Systems, Palo Alto, CA), 3D detector array devices are more suitable than simple 2D plane detectors in terms of the characteristics of arc therapy [6–9]. One limitation of conventional DQA processes is that they measure and analyze the dose in a phantom material, and not in the body of the patient. To overcome this problem, special tools have been developed to calculate the dose distribution in the bodies of patients using data measured in the DQA process [10,11].

There are two DQA methods used for dose calculation in the body. The first is to recalculate the dose in the computed tomography (CT) of the patient using the data measured by the detector arrays in conventional DQA processes. Typical tools that employ this method include 3DVH (SunNuclear, Melbourne, FL, USA), Compass (IBA, Schwarzenbruck, Germany), and Delta4DVH (ScandiDos, Uppsala, Sweden) [12–14].

The second is to calculate the dose in the body using the machine log file generated during treatment delivery. This approach does not require any dose measurement activity, and can save time by streamlining the workflow. To validate this approach in clinical applications, the accuracy of dose distribution calculated using the machine log file and that for doses calculated based on the measurement data have been studied and compared [15–17].

MobiusFX (Mobius Medical Systems, Houston, TX, USA) is a representative software package for calculating patient dose distribution based on a machine log file, and is an add-on software module to Mobius3D (Mobius Medical Systems, Houston, TX, USA). Mobius3D is a tool that can be used to verify doses calculated in TPS through an independent dose calculation method [18,19]. When the plan, structures, dose, and CT DICOM files are imported into Mobius3D from the TPS, the system automatically verifies the 3D dose of the plan in the CT of the patient using independently verified beam models and a collapsed-cone algorithm. During this process, MobiusFX automatically captures the log files from the treatment machine that contain the measured delivery data, and Mobius3D uses this data to calculate the 3D dose received by the patient positioned in that beam. This tool can be used in the DQA process before treatment delivery, and can also be applied to verify that the correct dose is delivered to the patient in each fraction.

The approach of DQA using a log file is fundamentally different than that of conventional IMRT DQA, and there is much debate regarding the accuracy of replacing the conventional DQA method based on the measured dose with this novel method [20,21]. Although MobiusFX has been studied in terms of its usefulness and accuracy, its use is limited to the evaluation of mechanical errors [22]. Consequently, more studies are required to determine its efficacy for evaluating various factors that may arise during treatment, such as changes in the dose output.

This paper describes the dosimetric evaluation of MobiusFX in various situations in order to determine the accuracy of the tool when applied to a RapidArc DQA process. During our study, the patient dose recalculated with MobiusFX was compared to the dose calculated using the 3DVH program, whose accuracy has been previously verified [23,24]. The dosimetric accuracy of each calculation tool was analyzed using gamma evaluation by varying the dose difference and distance-to-agreement criteria. In addition, we evaluated the variation in the calculated pass rate in the gamma evaluation and major dosimetric endpoints versus the increase in the position error and dosimetric output in the delivery of RapidArc treatment plans.

## Methods and materials

### Preparation of the RapidArc and DQA plans

A total of 30 RapidArc plans were prepared to evaluate the doses calculated in MobiusFX and 3DVH. The plans consisted of 10 prostate plans, 10 head-neck plans, and 10 chest plans. DQA plans corresponding to the prepared RapidArc plans were created with an ArcCHECK (SunNuclear, Melbourne, FL) diode detector array. All RapidArc and DQA plans were created using an Eclipse (Varian, Palo Alto, CA, USA) planning system. A clinical linear accelerator (LINAC), Novalis Tx (Varian, Palo Alto, CA, USA), was also used in this study.

### Dose recalculation in the body of the patient

The dosimetric data measured with an ArcCHECK during the delivery of the DQA plans was input to the 3DVH program to recalculate the dose inside the body of the patient. The MobiusFX tool requires access to the machine log file to recalculate the dose in the patient body in the DQA process. In this study, the machine log file was generated during the delivery of the original plan without the patient in QA mode.

### Comparison of the results from MobiusFX and3DVH

**Fig 1:**
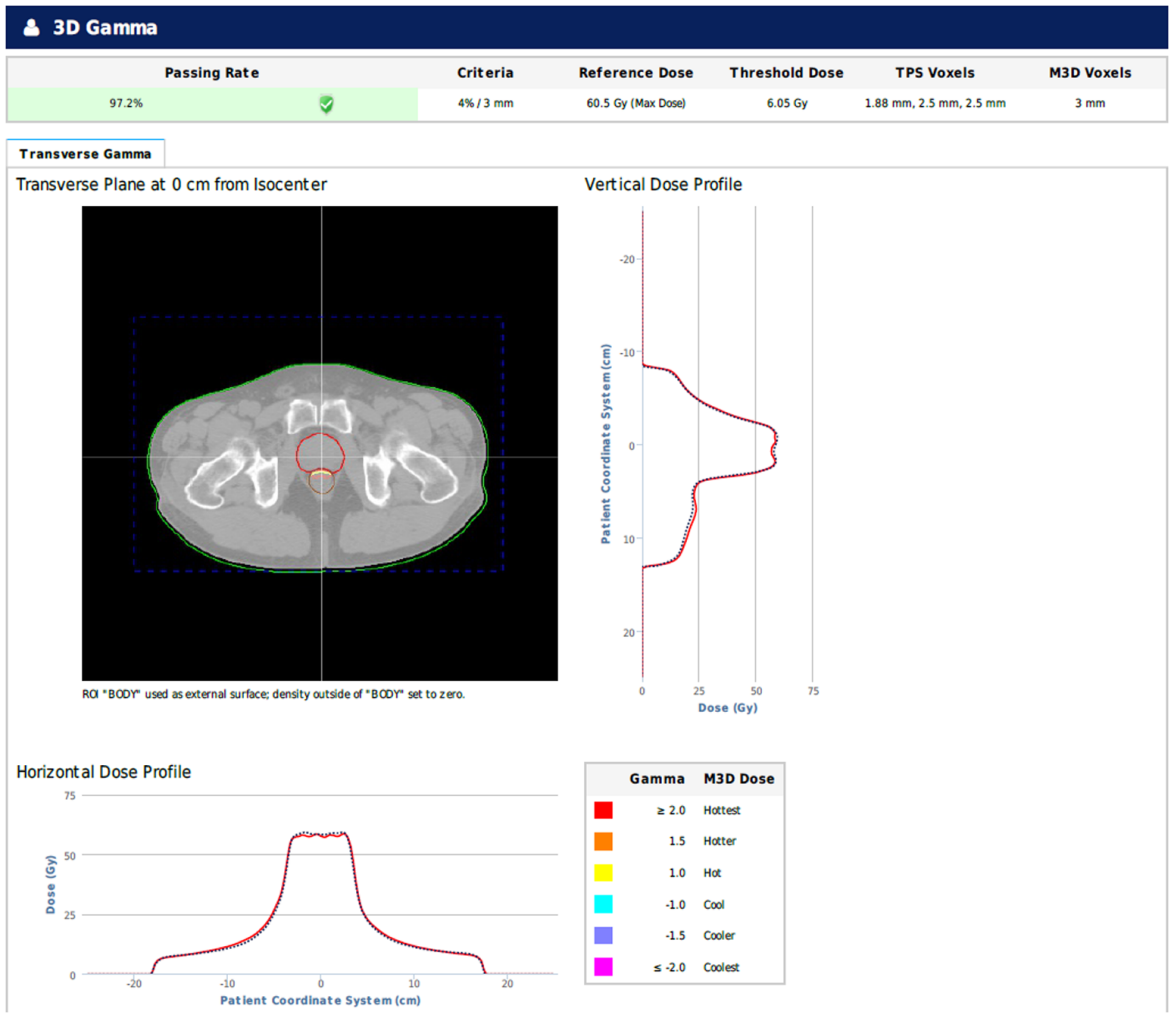
Example of gamma evaluation with the dose calculated in MobiusFX.

**Fig 2:**
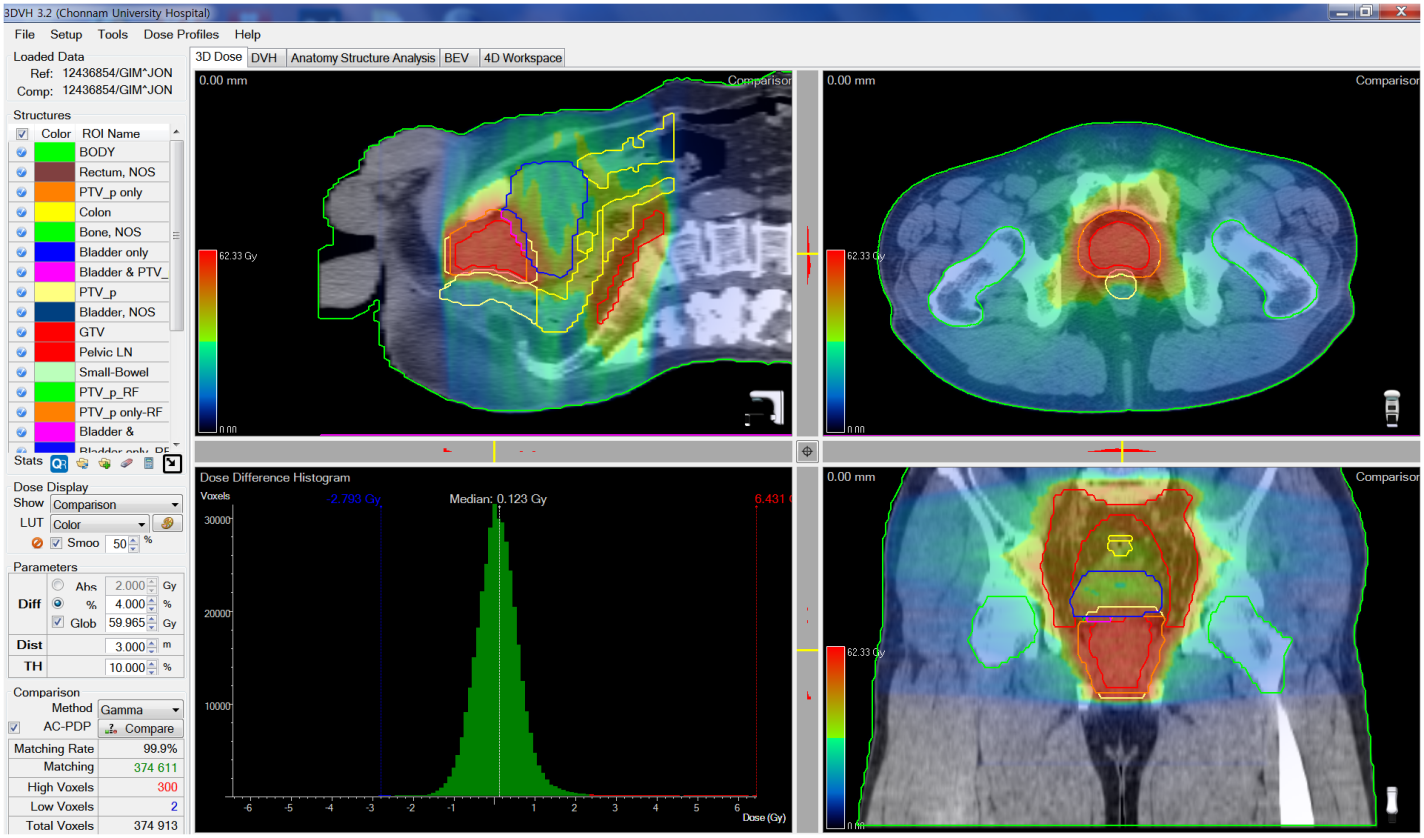
Example of gamma evaluation with the dose calculated in 3DVH.

The dosimetric discrepancies between the dose in the original plan and the recalculated dose using 3DVH and MobiusFX were analyzed with the gamma evaluation method. A comparison of the pass rates in the gamma evaluation between 3DVH and MobiusFX was performed based on three different values for the dose difference and distance-to-agreement: 3%–3 mm, 4%–3 mm, and 5%–3 mm, respectively. All gamma evaluations in this study were performed with a 10% threshold value. Figs 1 and 2 show an example of the evaluation results in MobiusFX and 3DVH.

The D_90%_, which is a typical dosimetric endpoint of a tumor target, was calculated and compared to the value in the original plan to compare the target dose coverage of the two methods. The difference in the D_90%_ was evaluated on a relative basis because the prescribed doses of the patients in this study were not same; thus, it would not have been correct to use the absolute value of the dose when comparing the deviation in the results.

Intentional false plans with position errors of 3 and 6 mm and increased doses of 3% and 6% compared to the values in the original plan were created to analyze the effect of position errors and increased dose outputs on the calculated dose in MobiusFX and 3DVH.

In the 3DVH calculation, the position error of 3 mm and 6 mm was applied by moving the ArcCHECK in the direction of the LINAC gantry. The origin value in the coordinate of the CT data was moved 3 mm and 6 mm in the cranial direction, and the same RapidArc plan was copied based on the moved CT data in order to apply the position error in MobiusFX calculation.

MobiusFX cannot directly evaluate the dosimetric output variation of the treatment machine because the machine log file does not contain the output information. Therefore, the monitor unit was changed to simulate the dosimetric output variation. The increased dose output was realized by normalizing the values at 97% and 94% compared with the original value at 100% in both 3DVH and MobiusFX.

The error plan was created with the same name as the original plan and was delivered in order to acquire the machine log file to calculate the dose under the error conditions using MobiusFX. After the error analysis, the plans in MobiusFX were deleted and the original plan was again exported to MobiusFX. Another error plan was created with the same name as the original plan and the delivery and error analysis process was repeated.

**Fig 3:**
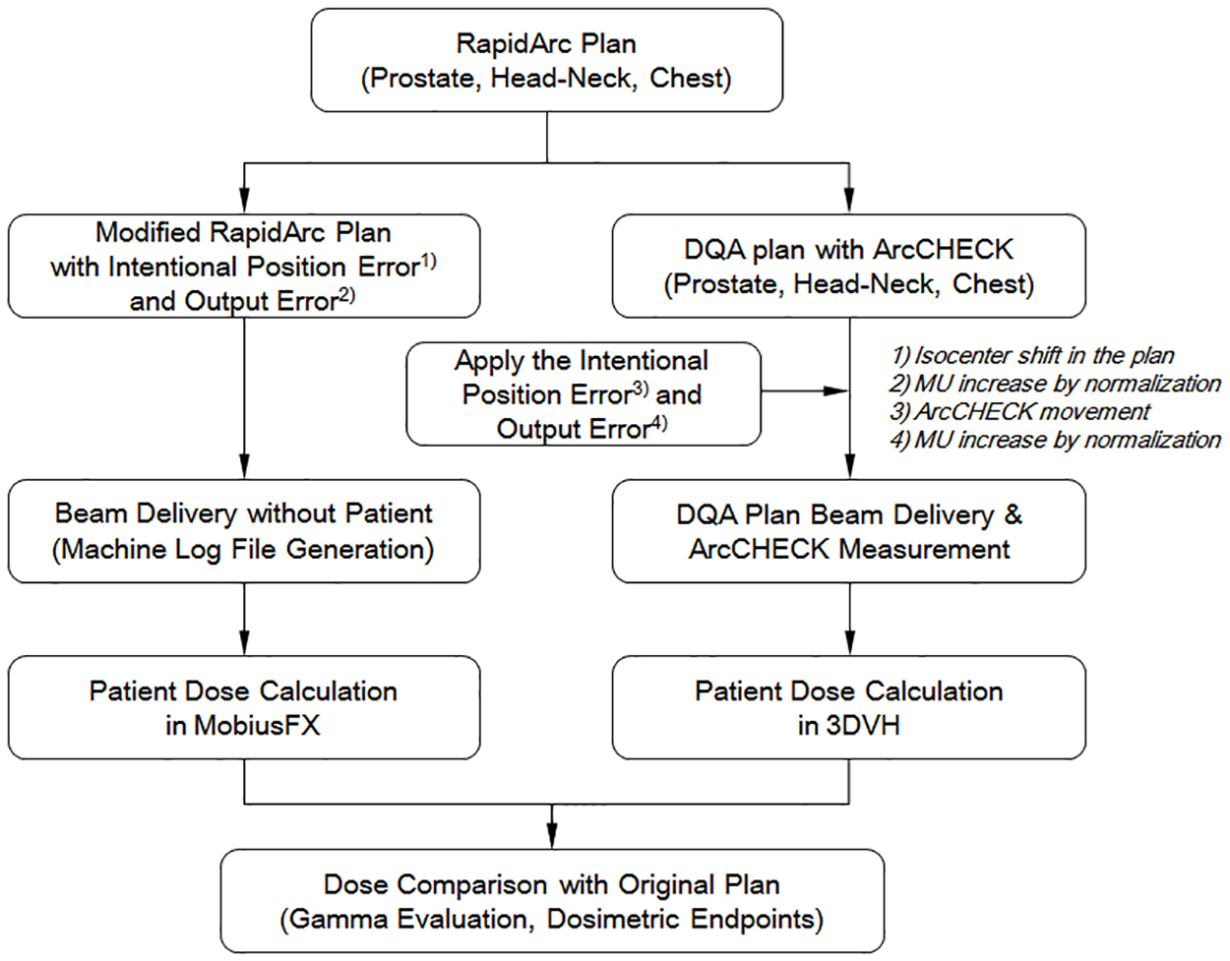
Process diagram for the dosimetric analysis MobiusFX and 3DVH in the simulated error condition of position and output.

Nine plans (3 prostate plans, 3 head-neck plans, and 3 chest plans) out of the 30 prepared RapidArc plans were selected to evaluate the calculated dose in the error conditions. Fig 3 shows the overall process for the dosimetric analysis of MobiusFX and 3DVH for the position and output simulated error conditions.

The error was evaluated according to the pass rate calculated using the gamma evaluation method with a 3% dose difference and 3-mm distance-to-agreement criteria. The gamma evaluation was performed in two structural volumes, which were a tumor target and an entire body corresponding to the irradiated volume within the dose calculation region.

The variation of the dosimetric endpoints in the error condition were also evaluated. D_90%_ was evaluated as a typical dosimetric endpoint of a tumor target, and D_max_ and D_mean_ were evaluated as major dosimetric endpoints in the OAR.

## Results

The pass rates calculated in the gamma evaluation between the dose in the original plan and the dose recalculated in MobiusFX and 3DVH are shown in Tables 1, 2, and 3.

**Table 1:** The pass rate calculated in the gamma evaluation of the prostate RapidArc plan calculated according to the criteria of dose difference and distance-to-agreement.

Patient	MobiusFX			3DVH		
	5%–3 mm	4%–3 mm	3%–3 mm	5%–3 mm	4%–3 mm	3%–3 mm
P1	99.2%	98.0%	95.1%	99.9%	99.8%	99.3%
P2	99.3%	98.6%	95.4%	99.8%	99.6%	99.0%
P3	99.8%	99.4%	96.4%	99.8%	99.6%	99.0%
P4	97.7%	94.8%	87.6%	99.9%	99.7%	98.7%
P5	99.1%	98.4%	96.7%	100.0%	99.9%	99.8%
P6	99.5%	98.5%	95.7%	99.9%	99.8%	99.5%
P7	96.4%	91.3%	84.5%	99.8%	99.8%	98.6%
P8	98.7%	97.2%	93.0%	99.9%	99.8%	99.5%
P9	100.0%	100.0%	99.9%	100.0%	100.0%	99.8%
P10	98.9%	97.2%	91.2%	100.0%	99.9%	99.7%
Average	98.9±1.1%	97.3±2.6%	93.6±4.6%	99.9±0.1%	99.8±0.1%	99.3±0.4%

**Table 2:** The pass rate calculated in the gamma evaluation of the head and neck RapidArc plan calculated according to the criteria of dose difference and distance-to-agreement.

Patient	MobiusFX			3DVH		
	5%–3 mm	4%–3 mm	3%–3 mm	5%–3 mm	4%–3 mm	3%–3 mm
H1	98.6%	97.9%	96.7%	100.0%	100.0%	99.9%
H2	98.3%	96.8%	94.2%	100.0%	100.0%	99.8%
H3	99.7%	99.1%	97.2%	100.0%	99.9%	99.7%
H4	99.7%	99.3%	98.5%	100.0%	99.9%	99.7%
H5	99.6%	99.2%	98.7%	100.0%	99.9%	99.1%
H6	96.6%	95.0%	92.6%	99.8%	99.7%	99.3%
H7	98.8%	96.4%	96.0%	100.0%	99.9%	99.8%
H8	95.5%	99.0%	98.2%	99.9%	99.8%	99.7%
H9	99.9%	99.8%	99.5%	100.0%	100.0%	99.9%
H10	99.3%	98.4%	97.4%	99.8%	99.5%	98.6%
Average	98.6±1.5%	98.1±1.6%	96.9±2.1%	100.0±0.1%	99.9±0.2%	99.6±0.4%

**Table 3:** The pass rate calculated in the gamma evaluation of the chest RapidArc plan calculated according to the criteria of dose difference and distance-to-agreement.

Patient	MobiusFX			3DVH		
	5%–3 mm	4%–3 mm	3%–3 mm	5%–3 mm	4%–3 mm	3%–3 mm
C1	98.9%	98.4%	97.3%	100.0%	99.9%	99.7%
C2	100.0%	99.5%	96.6%	100.0%	100.0%	99.9%
C3	99.3%	98.5%	96.7%	100.0%	99.9%	99.8%
C4	99.1%	97.5%	93.6%	100.0%	99.9%	99.8%
C5	99.8%	99.7%	99.1%	100.0%	100.0%	99.8%
C6	99.9%	99.7%	99.3%	100.0%	100.0%	99.9%
C7	99.5%	99.0%	97.9%	100.0%	99.9%	99.7%
C8	100.0%	99.9%	99.6%	100.0%	100.0%	100.0%
C9	100.0%	99.9%	99.7%	100.0%	100.0%	99.9%
C10	99.3%	98.8%	97.1%	100.0%	100.0%	99.9%
Average	99.6±0.4%	99.1±0.8%	97.7±1.9%	100.0±0.0%	100.0±0.1%	99.8±0.1%

The average pass rates in the prostate RapidArc plans were 98.9%, 97.3%, and 93.6% for the 5%–3 mm, 4%–3 mm, and 3%–3 mm criteria, respectively, when MobiusFX was used. The average pass rates were 99.9%, 99.8%, and 99.3% for the 5%–3 mm, 4%–3 mm, and 3%–3 mm criteria, respectively, when 3DVH was used.

The average pass rates in the head and neck RapidArc plans were 98.6%, 98.1%, and 96.9%for the 5%–3 mm, 4%–3 mm, and 3%–3 mm criteria, respectively, when MobiusFX was used. The average pass rates were 100.0%, 99.9%, and 99.6% for the 5%–3 mm, 4%-3 mm, and 3%–3 mm criteria, respectively, when 3DVH was used.

The average pass rates in the chest RapidArc plans were 99.6%, 99.1%, and 97.7% for the 5%–3 mm, 4%–3 mm, and 3%–3 mm criteria, respectively, when MobiusFX was used. The average pass rates were 100.0%, 100.0%, and 99.8% for the 5%–3 mm, 4%–3 mm, and 3%–3 mm criteria, respectively, when 3DVH was used.

In all cases, both methods showed suitable pass rates for IMRT DQA, but the pass rates in 3DVH were slightly higher than the values in MobiusFX, which showed more dose similarity with the original plan in 3DVH. MobiusFX exhibited increased sensitivity as the gamma evaluation criteria were strengthened, which was similar to the results of Au et al [22].

The calculated differences of D_90%_, which is a typical dosimetric endpoint of a tumor target, compared with the values in the original plan are shown in Table 4. The average differences were 0.85%, -3.50%, and 2.58% in the prostate, head and neck, and chest, respectively, when MobiusFX was used, and the average differences were 0.61%, 0.40%, and 0.69% in the prostate, head and neck, and chest, respectively, when 3DVH was used. The calculated differences in 3DVH were less than 1% on average, and MobiusFX exhibited differences of less than 3%, which was larger than those in 3DVH. As shown in the gamma evaluation results, the calculated D_90%_ of the tumor target in 3DVH was more similar to the original plan than was the dose calculated by MobiusFX.

**Table 4:** The relative differences of D_90%_ of tumor target in MobiusFX and 3DVH compared with the values in the original plan.

Patient No.	Prostate		Head & Neck		Chest	
	MobiusFX	3DVH	MobiusFX	3DVH	MobiusFX	3DVH
1	1.02%	0.68%	-3.39%	-0.72%	-0.01%	2.40%
2	-0.19%	1.21%	-5.36%	-0.95%	-1.33%	-0.42%
3	0.36%	1.03%	-3.08%	-0.29%	-2.32%	1.97%
4	2.80%	-0.88%	-4.89%	1.24%	-3.59%	0.40%
5	-0.87%	-0.09%	-2.46%	-1.42%	-2.48%	-0.14%
6	1.06%	0.63%	-8.55%	0.45%	-1.78%	-0.41%
7	0.51%	1.11%	-2.68%	-0.63%	-6.63%	1.22%
8	2.09%	0.81%	-0.02%	0.53%	-3.50%	1.45%
9	0.79%	0.59%	-3.14%	-1.70%	-0.38%	0.60%
10	0.96%	1.03%	-1.44%	-0.55%	-3.81%	-0.20%
Average	0.85±1.04%	0.61±0.64%	-3.50±2.34%	0.40±0.91%	-2.58±1.93%	0.69±1.02%

The results of analyzing the difference in the calculated dose distribution caused by the introduction of virtual position and output errors relative to the original plan for each method are as follows.

The average pass rates for the 6 mm position error in the case of MobiusFX were 83.0% inside the entire body and 93.9% inside the tumor target. The average pass rates for 3DVH for the same position error were 64.0% inside the entire body and 64.3% inside the tumor target.

The average pass rates for the 3mm position error in the case of MobiusFX were 83.0% inside the entire body and 93.8% inside the tumor target. The average pass rates in the case of 3DVH for the same position error were 87.2% inside the entire body and 80.2% inside the tumor target.

**Fig 4:**
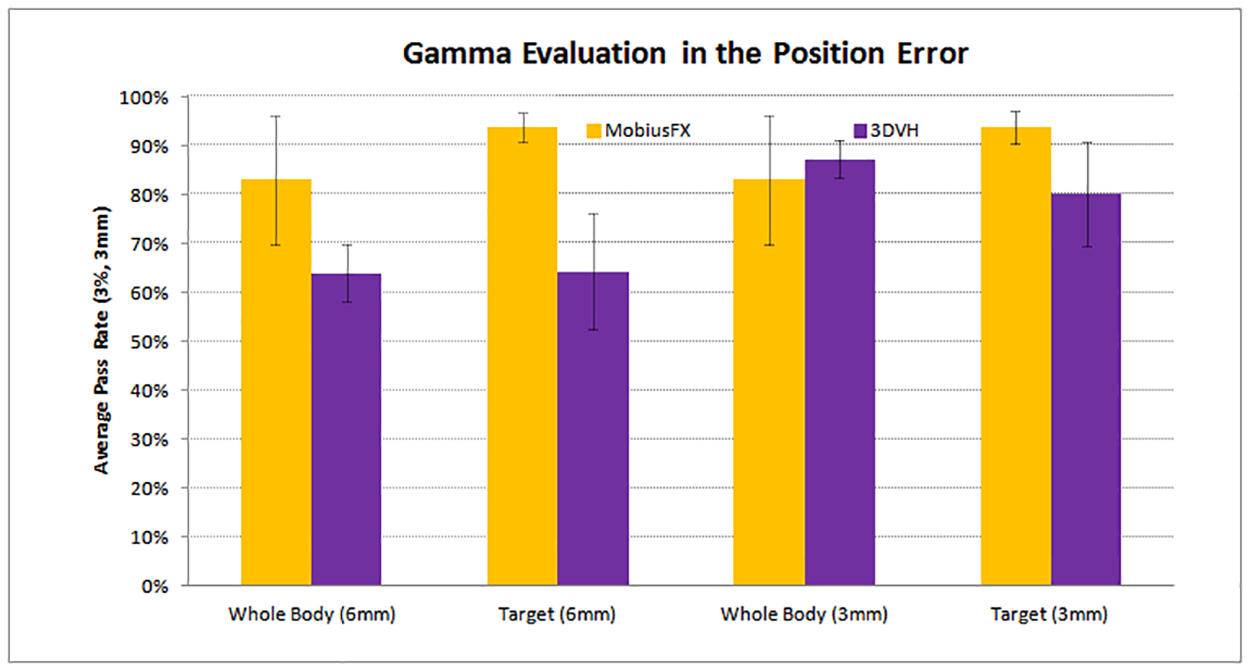
The comparison of average pass rate between MobiusFX and 3DVH in the position error.

A comparison of the average pass rate between MobiusFX and 3DVH in the position error is shown in Fig 4. In the case of the position error, 3DVH showed a lower pass rate than MobiusFX, which indicates that 3DVH is more sensitive to position error in dose calculations.

The average pass rates in MobiusFX for the 6% increased dose error were 91.3% inside the entire body and 29.2% inside the tumor target. The average pass rates in the case of 3DVH for the same output error were 82.2% inside the entire body and 23.8% inside the tumor target.

The average pass rates in MobiusFX for the 3% increased dose error were 96.6% inside the entire body and 74.0% inside the tumor target. The average pass rates in the case of 3DVH for the same output error were 94.7% inside the entire body and 61.5% inside the tumor target.

**Fig 5:**
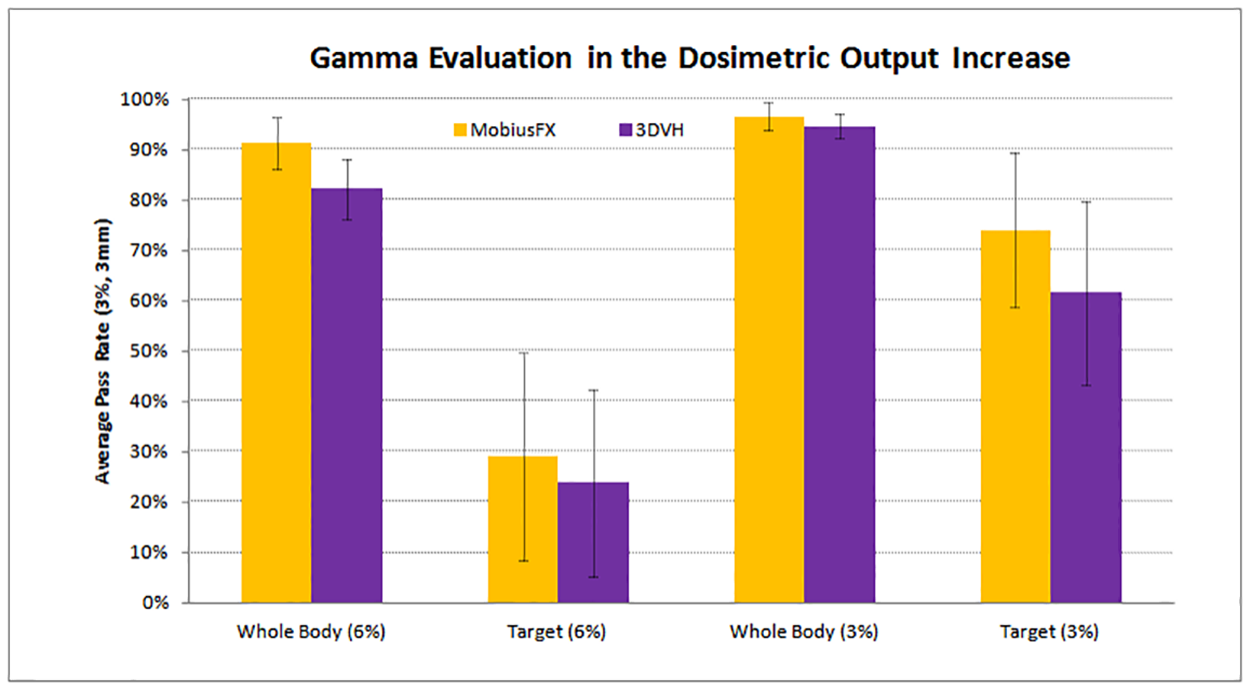
The comparison of average pass rate between MobiusFX and 3DVH in the dosimetric output increase.

Fig 5 shows a comparison of the average pass rate between MobiusFX and 3DVH versus the dosimetric output error. Although 3DVH showed a lower pass rate, the difference with MobiusFX was not large compared to the results for the position error. In addition, the pass rate at the target was more sensitive to the output error than the position error.

Although there was a considerable dose error compared with the original plan when the virtual output increase was applied to MobiusFX, there was no way to include information regarding the output change in the machine log file used in actual clinical applications. This restriction may be regarded as one of the major disadvantages of MobiusFX. In contrast, 3DVH used ArcCHECK dose measurement results, which were able to detect the output change. This difference could be considered more useful in terms of DQA.

The variation of the dosimetric endpoints in the error condition is shown in Tables 5–10.

**Table 5:** The relative differences of the dosimetric endpoints of prostate cases between the calculated values in MobiusFX under the intentional error conditions and the calculated values in the original plan.

Patient	Dosimetric Endpoints	Original Plan Delivery	Position Error		Output Increase	
			6 mm	3 mm	6%	3%
P1						
	Target					
	D_90%_	1.02%	-1.83%	-1.98%	5.28%	1.99%
	Rectum					
	D_max_	-1.56%	0.01%	-0.02%	8.83%	5.43%
	D_mean_	-4.67%	-1.60%	-1.71%	6.65%	3.32%
	Bladder					
	D_max_	-1.03%	-1.40%	-1.61%	7.64%	4.28%
	D_mean_	-1.75%	-1.41%	-1.26%	7.41%	4.05%
P2						
	Target					
	D_90%_	-0.19%	-0.62%	0.66%	5.50%	4.50%
	Rectum					
	D_max_	-0.27%	1.69%	1.98%	8.92%	5.55%
	D_mean_	-7.67%	-5.95%	-6.03%	2.92%	-0.26%
	Bladder					
	D_max_	0.73%	4.80%	4.94%	9.05%	5.68%
	D_mean_	-0.24%	1.06%	1.06%	8.67%	5.31%
P3						
	Target					
	D_90%_	0.36%	-1.76%	-1.78%	4.79%	1.52%
	Rectum					
	D_max_	-1.03%	1.06%	0.56%	9.07%	5.66%
	D_mean_	-5.04%	-2.38%	-2.79%	5.70%	2.39%
	Bladder					
	D_max_	0.75%	-0.67%	-0.48%	8.75%	5.35%
	D_mean_	-5.00%	-2.32%	-2.48%	4.39%	1.12%

**Table 6:** The relative differences of the dosimetric endpoints of prostate cases between the calculated values in 3DVH under the intentional error conditions and the calculated values in the original plan.

Patient	Dosimetric Endpoints	Original Plan Delivery	Position Error		Output Increase	
			6 mm	3 mm	6%	3%
P1						
	Target					
	D_90%_	0.68%	-1.47%	-1.49%	6.77%	3.73%
	Rectum					
	D_max_	2.70%	-4.23%	-3.87%	8.92%	5.81%
	D_mean_	-0.45%	-0.70%	-0.77%	5.59%	2.57%
	Bladder					
	D_max_	2.47%	-5.77%	-5.84%	8.68%	5.57%
	D_mean_	1.41%	-3.29%	-3.22%	7.56%	4.48%
P2						
	Target					
	D_90%_	1.21%	-1.33%	-1.31%	7.28%	4.11%
	Rectum					
	D_max_	5.20%	-5.10%	-5.03%	11.52%	8.36%
	D_mean_	0.54%	-1.57%	-1.55%	6.57%	3.53%
	Bladder					
	D_max_	6.06%	-6.13%	-5.65%	12.42%	9.24%
	D_mean_	1.53%	-2.67%	-2.64%	7.62%	4.58%
P3						
	Target					
	D_90%_	1.03%	-1.98%	-1.97%	7.15%	4.08%
	Rectum					
	D_max_	4.57%	-5.99%	-5.86%	10.90%	7.73%
	D_mean_	-1.20%	0.16%	0.01%	4.79%	1.79%
	Bladder					
	D_max_	6.52%	-7.00%	-7.93%	12.97%	9.75%
	D_mean_	2.10%	-3.27%	-3.32%	8.29%	5.19%

**Table 7:** The relative differences of the dosimetric endpoints of head and neck cases between the calculated values in MobiusFX under the intentional error conditions and the calculated values in the original plan.

Patient	Dosimetric Endpoints	Original Plan Delivery	Position Error		Output Increase	
			6 mm	3 mm	6%	3%
H1						
	Target					
	D_90%_	-3.39%	-2.73%	-2.79%	4.19%	0.92%
	Pituitary					
	D_max_	-11.33%	-8.29%	-8.36%	-3.25%	-6.27%
	D_mean_	-9.34%	-6.89%	-6.89%	-1.20%	-4.29%
	Brain Stem					
	D_max_	12.00%	16.81%	16.69%	23.84%	19.97%
	D_mean_	-6.92%	-3.67%	-3.75%	1.80%	-1.39%
	Eyeball					
	D_max_	-11.96%	-9.41%	-9.56%	-3.98%	-6.99%
	D_mean_	-9.71%	-6.72%	-6.52%	-0.76%	-3.86%
H2						
	Target					
	D_90%_	-5.36%	-3.27%	-3.37%	2.15%	-0.99%
	Pituitary					
	D_max_	-8.12%	-2.18%	-2.38%	2.36%	-0.81%
	D_mean_	-3.43%	2.81%	2.93%	7.26%	3.95%
	Brain Stem					
	D_max_	-2.69%	-0.21%	0.26%	5.40%	2.14%
	D_mean_	-5.26%	-2.26%	-1.96%	2.83%	-0.54%
	Eyeball					
	D_max_	10.67%	-4.60%	-3.87%	-0.79%	-3.87%
	D_mean_	-8.28%	-2.61%	-1.83%	2.81%	-0.37%
H3						
	Target					
	D_90%_	-3.08%	-1.50%	-1.45%	5.15%	1.90%
	Spinal Cord					
	D_max_	-3.09%	2.15%	1.99%	4.88%	1.64%
	D_mean_	-4.59%	-3.24%	-3.19%	3.56%	0.35%
	Parotid Gland					
	D_max_	-1.64%	1.42%	1.01%	6.64%	6.68%
	D_mean_	-9.24%	-5.23%	-5.12%	-0.36%	-3.27%
	Thyroid Gland					
	D_max_	-3.67%	2.23%	-2.14%	5.68%	2.41%
	D_mean_	-5.33%	-1.86%	-2.12%	4.42%	1.19%

**Table 8:** The relative differences of the dosimetric endpoints of head and neck cases between the calculated values in 3DVH under the intentional error conditions and the calculated values in the original plan.

Patient	Dosimetric Endpoints	Original Plan Delivery	Position Error		Output Increase	
			6 mm	3 mm	6%	3%
H1						
	Target					
	D_90%_	-0.72%	-0.22%	0.19%	5.29%	2.29%
	Pituitary					
	D_max_	-19.67%	-19.38%	-19.34%	-14.80%	-17.24%
	D_mean_	-16.40%	-15.76%	-15.73%	-11.33%	-13.86%
	Brain Stem					
	D_max_	-4.33%	-4.20%	-4.39%	1.47%	-1.43%
	D_mean_	-14.28%	-13.78%	-13.80%	-9.09%	-11.68%
	Eyeball					
	D_max_	-0.66%	2.13%	2.23%	5.36%	2.34%
	D_mean_	-7.09%	-6.54%	-6.54%	-1.86%	-3.76%
H2						
	Target					
	D_90%_	-0.95%	1.79%	-0.37%	4.99%	2.03%
	Pituitary					
	D_max_	-4.52%	-27.40%	-23.90%	1.21%	-1.66%
	D_mean_	-7.89%	-34.12%	-19.92%	-2.37%	-5.13%
	Brain Stem					
	D_max_	0.12%	-12.05%	-9.04%	6.13%	3.12%
	D_mean_	-1.31%	-8.76%	-5.43%	4.61%	1.65%
	Eyeball					
	D_max_	-4.02%	-6.85%	1.17%	1.74%	-1.14%
	D_mean_	-9.71%	-9.07%	-6.93%	-4.29%	-7.00%
H3						
	Target					
	D_90%_	-0.29%	-0.25%	-0.25%	5.70%	2.69%
	Spinal Cord					
	D_max_	7.78%	5.83%	5.84%	14.25%	11.02%
	D_mean_	-0.19%	-0.36%	-0.33%	5.80%	2.81%
	Parotid Gland					
	D_max_	-0.73%	-1.83%	-1.80%	5.23%	2.25%
	D_mean_	-3.93%	-3.73%	-3.73%	1.84%	-1.05%
	Thyroid Gland					
	D_max_	-0.10%	2.29%	2.29%	5.89%	2.90%
	D_mean_	-2.80%	-2.81%	-2.83%	3.03%	0.11%

**Table 9:** The relative differences of the dosimetric endpoints of chest cases between the calculated values in MobiusFX under the intentional error conditions and the calculated values in the original plan.

Patient	Dosimetric Endpoints	Original Plan Delivery	Position Error		Output Increase	
			6 mm	3 mm	6%	3%
C1						
	Target					
	D_90%_	-0.01%	-8.38%	-8.17%	3.27%	0.12%
	Esophagus					
	D_max_	0.81%	1.75%	1.04%	10.24%	6.76%
	D_mean_	-0.46%	2.20%	1.64%	9.71%	6.24%
	Spinal Cord					
	D_max_	-10.43%	-13.11%	-10.33%	-0.72%	-3.86%
	D_mean_	-3.12%	0.45%	-0.60%	8.90%	6.57%
	Lung					
	D_max_	-0.14%	1.12%	-0.13%	9.28%	5.83%
	D_mean_	-6.39%	-5.13%	-5.08%	3.09%	-0.17%
C2						
	Target					
	D_90%_	-1.33%	-0.96%	-1.20%	6.61%	3.14%
	Esophagus					
	D_max_	2.81%	0.18%	-0.11%	10.59%	10.59%
	D_mean_	-3.01%	-2.72%	-3.14%	5.73%	5.73%
	Spinal Cord					
	D_max_	-0.39%	-2.10%	-2.45%	7.22%	7.22%
	D_mean_	-9.86%	-9.41%	-9.72%	-1.71%	-1.71%
	Lung					
	D_max_	4.95%	3.16%	3.13%	13.10%	13.10%
	D_mean_	-16.52%	-14.74%	-14.89%	-8.54%	-8.53%
C3						
	Target					
	D_90%_	-2.32%	-2.87%	-2.84%	2.09%	-1.06%
	Esophagus					
	D_max_	3.29%	4.86%	4.75%	11.09%	7.66%
	D_mean_	-2.81%	0.78%	0.76%	6.00%	2.72%
	Spinal Cord					
	D_max_	0.60%	2.52%	2.55%	8.15%	4.81%
	D_mean_	2.01%	4.91%	4.88%	10.45%	7.03%
	Lung					
	D_max_	-2.12%	-0.27%	-0.21%	3.50%	2.29%
	D_mean_	-6.92%	-3.13%	-3.22%	0.21%	-1.22%

**Table 10:** The relative differences of the dosimetric endpoints of chest cases between the calculated values in 3DVH under the intentional error conditions and the calculated values in the original plan.

Patient	Dosimetric Endpoints	Original Plan Delivery	Position Error		Output Increase	
			6 mm	3 mm	6%	3%
C1						
	Target					
	D_90%_	2.40%	4.72%	4.94%	8.67%	5.53%
	Esophagus					
	D_max_	-1.80%	0.77%	1.66%	4.22%	1.21%
	D_mean_	-0.77%	-0.63%	0.56%	5.00%	2.27%
	Spinal Cord					
	D_max_	-3.84%	-1.10%	-3.79%	2.05%	-0.90%
	D_mean_	-8.69%	-6.62%	-5.38%	-3.09%	-5.89%
	Lung					
	D_max_	3.56%	4.85%	5.12%	9.90%	6.73%
	D_mean_	-0.47%	1.44%	1.38%	5.62%	2.58%
C2						
	Target					
	D_90%_	-0.42%	5.23%	5.23%	6.07%	2.92%
	Esophagus					
	D_max_	0.39%	6.14%	6.21%	6.73%	3.56%
	D_mean_	-0.90%	5.19%	5.22%	5.36%	2.23%
	Spinal Cord					
	D_max_	-0.70%	3.61%	3.80%	5.57%	2.43%
	D_mean_	-1.59%	2.94%	3.00%	4.62%	1.52%
	Lung					
	D_max_	1.98%	6.82%	6.86%	8.42%	5.19%
	D_mean_	-1.09%	5.56%	3.52%	5.15%	2.03%
C3						
	Target					
	D_90%_	1.97%	12.91%	3.46%	8.09%	5.04%
	Esophagus					
	D_max_	-0.95%	-3.52%	-0.80%	5.00%	2.03%
	D_mean_	-1.00%	-1.02%	-1.37%	4.94%	1.97%
	Spinal Cord					
	D_max_	-1.77%	-0.89%	-1.36%	4.12%	1.18%
	D_mean_	-3.19%	-1.21%	-2.20%	2.61%	-0.29%
	Lung					
	D_max_	6.02%	5.07%	5.30%	12.38%	9.19%
	D_mean_	-0.79%	-4.29%	-2.58%	5.16%	2.18%

Both MobiusFX and 3DVH produced higher endpoint value for the tumor target and OARs when the output was increased relative to the original plan. When the position error was applied, the variation in the endpoint value changed depending on the location and shape of the structures in the dose distribution region, and no correlation was observed between the results of MobiusFX and 3DVH. Based on these results, we conclude that evaluating the dose error based only on the pass rate in the gamma evaluation does not consider important changes in the dose distribution value in the actual tumor target and OAR. Therefore, it is necessary to also analyze the various dosimetric endpoints.

## Discussion

The gamma evaluation in this study between the dose in an original plan and the recalculated dose in the DQA process showed lower pass rates in the MobiusFX evaluation compared with the values calculated in 3DVH. The calculated dose between MobiusFX and 3DVH was not the same due to differences in the method of calculation. MobiusFX uses the collapsed convolution/superposition calculation algorithm and 3DVH applies the ArcCHECK planned dose perturbation method. Although dose differences due to differences in the calculation algorithm were inevitable, MobiusFX produced a significantly lower pass rate than 3DVH as the gamma evaluation criteria were tightened. The general criteria used in the gamma evaluation of the conventional DQA process, which were 3%-3 mm, should be used with caution in MobiusFX in order to realize a pass rate of more than 95%, which is considered to be the minimum required pass rate for the IMRT DQA process to be considered accurate. Consequently, the gamma evaluation criteria used in MobiusFX should be reviewed before clinical application.

In the gamma evaluation between the dose in an original plan and the dose calculated in the beam delivery with an intended error, MobiusFX produced a higher pass rate than 3DVH in most error cases. In 3DVH, a lower pass rate was calculated for the 6 mm error compared to that for a 3 mm error, while there was little difference in the pass rate of MobiusFX for 6 mm and 3 mm errors. This implies that 3DVH is more sensitive to position errors than MobiusFX, and mechanical errors, such as a MLC leaf position error, has less effect on the MobiusFX calculation than that for 3DVH.

When the increased dosimetric output was simulated, a higher pass rate was observed in the gamma evaluation for MobiusFX compared with that for 3DVH, and the difference in the pass rates were smaller than those for the position error. MobiusFX exhibited a lower pass rate inside the tumor target and a higher pass rate inside the entire body relative to the pass rate in the case of a position error. The results show that the increased gamma value due to a 3% higher dose inside the tumor target that is intensively irradiated with a prescribed dose is more sensitive than that for an increase due to a position error. The trend of the pass rate changes due to an increased dosimetric output was similar to that in the case of 3DVH. The results show that an output error of the clinical LINAC machine can induce greater dosimetric errors in the tumor target and surrounding OARs during a RapidArc treatment compared to that for mechanical position errors.

In the case of position errors, the dose distribution changes of a tumor target and OARs showed various patterns depending on the shape of each structure and its position in the dose distribution, as can be seen in the analysis of the change of dosimetric endpoints. Therefore, a dose volume histogram (DVH) analysis of the tumor target and OAR based on the calculated dose distribution should be done together rather than merely evaluating the gamma evaluation results in the DQA process using MobiusFX and 3DVH to better understand the influence on tumor control and OAR risk.

The use of IMRT DQA in patient geometries using treatment log files is still controversial in terms of accuracy and whether it can replace conventional measurement based DQA [25]. MobiusFX recalculates the dose inside the patient with an independent dose calculation algorithm using a log file generated during a treatment process, although it cannot consider output variations caused by the treatment LINAC machine. As can be seen in the results of this study, the dosimetric error inside the tumor target compared with that in the plan was greater when the output increased than in the position error. Although output variation should be considered in the IMRT DQA process, the machine log file based DQA tool, such as MobiusFX, cannot evaluate output variations, which prevents its use as an ideal stand-alone IMRT DQA tool. The conventional IMRT DQA process, which is based on a measured dose in a phantom, can still be considered as the proper method to verify dosimetric accuracy considering the possible output variation in the treatment machine. At the same time, it is reasonable to use MobiusFX as a supplementary DQA tool to verify the mechanical accuracy in the daily IMRT treatment process in addition to conventional dosimetric measurement based DQA processes.

## Conclusions

In this study, the evaluation results of MobiusFX, which is a representative machine log file based IMRT DQA tool, showed similar dosimetric results as those exhibited by 3DVH, a conventional measurement based DQA program, in the presence of variable error conditions. These results confirm that MobiusFX has sufficient accuracy and is effective for IMRT DQA. However, a limitation of MobiusFX is that it cannot verify the error due to the output variation of the treatment machine. Consequently, a conventional dosimetric measurement baseline IMRT DQA should be performed before starting patient treatment. Based on these results, it is reasonable to use MobiusFX as a supplementary DQA tool to identify mechanical errors in the regular treatment process.
